# Chirality-Dependent Anti-Inflammatory Effect of Glutathione after Spinal Cord Injury in an Animal Model

**DOI:** 10.3390/ph14080792

**Published:** 2021-08-12

**Authors:** Seong-Jun Kim, Wan-Kyu Ko, Gong-Ho Han, Daye Lee, Yuhan Lee, Seung-Hun Sheen, Je-Beom Hong, Seil Sohn

**Affiliations:** 1 Department of Biomedical Science, CHA University, Seongnam-si 13493, Korea; ksj987456@chauniv.ac.kr (S.-J.K.); wankyu@chauniv.ac.kr (W.-K.K.); hgh429@chauniv.ac.kr (G.-H.H.); day03@chauniv.ac.kr (D.L.); 2 Department of Medicine, Brigham and Women’s Hospital, Harvard Medical School, Boston, MA 02115, USA; ylee21@bwh.harvard.edu; 3 Department of Neurosurgery, CHA Bundang Medical Center, Seongnam-si 13496, Korea; nssheen@cha.ac.kr; 4 Department of Neurosurgery, Kangbuk Samsung Hospital, Sungkyunkwan University School of Medicine, Seoul 03181, Korea; jebeomhong@gmail.com

**Keywords:** spinal cord injuries, chirality, glutathione, neuroinflammation, glial scars

## Abstract

Neuroinflammation forms a glial scar following a spinal cord injury (SCI). The injured axon cannot regenerate across the scar, suggesting permanent paraplegia. Molecular chirality can show an entirely different bio-function by means of chiral-specific interaction. In this study, we report that d-chiral glutathione (D-GSH) suppresses the inflammatory response after SCI and leads to axon regeneration of the injured spinal cord to a greater extent than l-chiral glutathione (L-GSH). After SCI, axon regrowth in D-GSH-treated rats was significantly increased compared with that in L-GSH-treated rats (*** *p* < 0.001). Secondary damage and motor function were significantly improved in D-GSH-treated rats compared with those outcomes in L-GSH-treated rats (** *p* < 0.01). Moreover, D-GSH significantly decreased pro-inflammatory cytokines and glial fibrillary acidic protein (GFAP) via inhibition of the mitogen-activated protein kinase (MAPK) signaling pathway compared with L-GSH (*** *p* < 0.001). In primary cultured macrophages, we found that D-GSH undergoes more intracellular interaction with activated macrophages than L-GSH (*** *p* < 0.001). These findings reveal a potential new regenerative function of chiral GSH in SCI and suggest that chiral GSH has therapeutic potential as a treatment of other diseases.

## 1. Introduction

Approximately 17,730 new cases of spinal cord injuries (SCI) occur for every year in the United States alone [1]. SCI triggers a complex local inflammatory response, which is a critical pathophysiological process related to secondary damage after the initial SCI. Inflammation is a major feature of secondary damage and is excessively induced at a very early stage after an acute injury [2,3]. The excessive inflammatory response induces glial scar formation, which can cause permanent paraplegia by disturbing axon contact. High doses of methylprednisolone, a corticosteroid anti-inflammatory drug, have been used worldwide to treat acute SCI patients [4]. However, there has been considerable debate over its use due to its adverse side effects [5,6,7].

Chirality is a unique property of molecules with identical elemental compositions but that are non-superimposable [8,9]. Materials in the environment have one of two naturally selected enantiomeric forms. For example, amino acids in proteins show mostly l-form, whereas sugar shows d-form. In biological systems, the chiral property can show entirely different functions due to chiral-specific interactions [10,11].

Glutathione (GSH) is a thiol-containing tripeptide that consists of cysteine, glutamic acid, and glycine. GSH has two enantiomer forms because cysteine and glutamic acid contain at least one chiral center. Previous studies have reported that GSH plays an anti-inflammatory role in in vivo models [12,13]. However, all of the studies examined were only based on the L-chiral form of GSH (L-GSH). To the best of our knowledge, the d-chiral form of GSH (D-GSH) has not been investigated as to whether it affects SCI outcomes. Therefore, in this study, we investigated whether the chiral identity of GSH affects anti-inflammatory function in an SCI animal model.

## 2. Results

### 2.1. Characterization of Chiral Gshs

**Figure 1:**
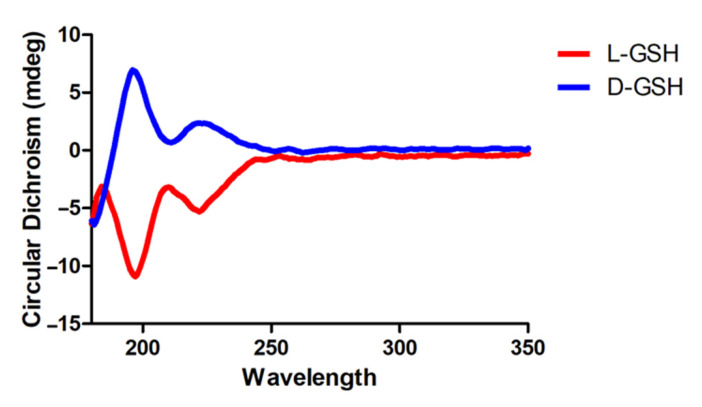
Circular dichroism (CD) spectra of L-chiral form of GSH (L-GSH; red) and D-chiral form of GSH (D-GSH; blue).

To investigate the optical activity of chiral GSHs, we dissolved L- or D-GSH in distilled water and measured the optical activity using circular dichroism (CD) spectroscopy. As a result, L-GSH showed a strong negative peak, whereas D-GSH showed a strong positive signal at around 190 nm (Figure 1). This indicates symmetrical CD signals and a distinct Cotton effect at around 190 nm [14]. Litman and Schellman reported that the optical rotatory dispersion of peptides was correlated with the conformational and structural features of a molecule [15]. Taken together, we confirmed that L- and D-GSH had opposite chirality.

### 2.2. Chiral GSH Decreased the Production of Inflammatory Cytokines after SCI

**Figure 2:**
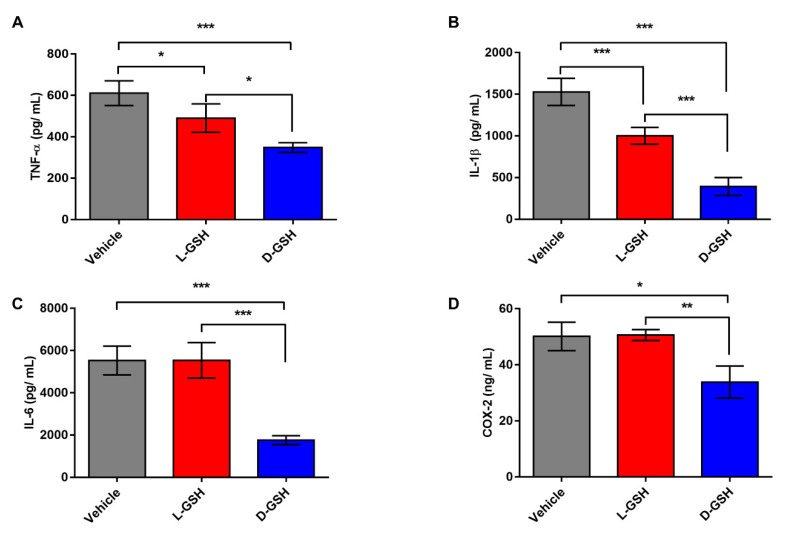
The protein secretion levels in the injured spinal cord segments of the vehicle, L-GSH, and D-GSH groups after SCI. Quantification of (**A**) Tumor necrosis factor-α (TNF-α), (**B**) interleukin (IL)-1β, (**C**) IL-6, and (**D**) cyclooxygenase-2 (COX-2). Results are the mean ± SEM; * *p* < 0.05, ** *p* < 0.01, and *** *p* < 0.001; one-way ANOVA with Tukey post hoc test.

To investigate the different anti-inflammatory effects by chirality of two enantiomers of GSH, we analyzed immune responses in injured spinal cords of vehicle-, L-GSH, and D-GSH-treated rats after SCI. Tumor necrosis factor-α (TNF-α) and interleukin (IL)-1β in the L-GSH group were significantly lower than those in the vehicle group (Figure 2A,B, * *p* < 0.05). However, IL-6 and cyclooxygenase-2 (COX-2) levels in the L-GSH group were not significantly decreased compared with those in the vehicle group (Figure 2C,D). On the other hand, TNF-α level in the D-GSH group was significantly suppressed compared with that in the L-GSH group (* *p* < 0.05). Moreover, this tendency was also found in other inflammatory cytokines, in this case IL-1β, IL-6, and COX-2 (** *p* < 0.01, *** *p* < 0.001). Pro-inflammatory cytokines including TNF-α, IL-1β, IL-6, and COX-2 play crucial roles in the immune system. Our results show that the pro-inflammatory cytokines levels in the D-GSH group were significantly decreased compared with those in the L-GSH group, suggesting that D-GSH efficiently inhibits activating immune response more than L-GSH.

### 2.3. D-GSH Inhibited Glial Scar Formation after SCI

**Figure 3:**
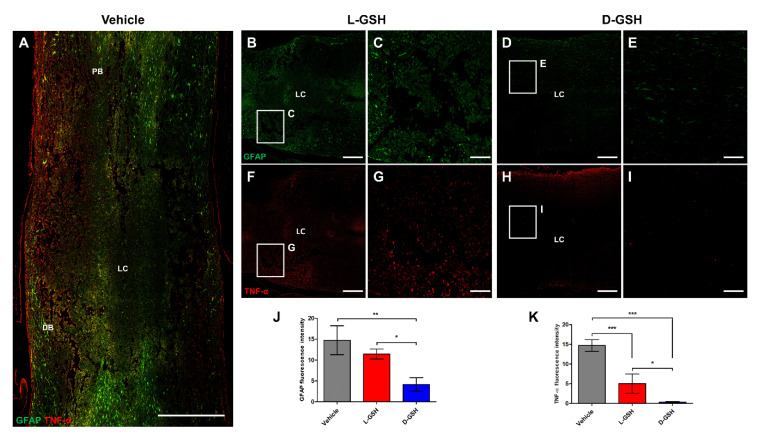
Immunofluorescence analysis in composite tiled scans of sagittal sections stained for glial scar (anti-glia fibrillary acidic protein, GFAP, Green), inflammatory cytokine (anti-tumor necrosis factor-α, TNF-α, Red). Proximal border (PB) and distal border (DB) away from the lesion center (LC). (**A**) Representative merged image for GFAP and TNF-α in the vehicle group. (**B**) Representative image for GFAP in the L-GSH group. (**C**) Higher magnification of the proximal border (boxed area) from (**B**). (**D**) Representative image for GFAP in the D-GSH group. (**E**) Higher magnification of the proximal border (boxed area) from (**D**). (**F**) Representative image for TNF-α in the L-GSH group. (**G**) Higher magnification of the proximal border (boxed area) from (**F**). (**H**) Representative image for TNF-α in the D-GSH group. (**I**) Higher magnification of the proximal border (boxed area) from (**H**). Scale bar, 500 μm (**A**,**B**,**D**,**F**,**H**) and 100 μm (**C**,**E**,**G**,**I**). Quantitative analyses of the fluorescence intensity for (**J**) GFAP and (**K**) TNF-α. Results are the mean ± standard error of the mean (SEM); * *p* < 0.05, ** *p* < 0.01, and *** *p* < 0.001; one-way ANOVA with Tukey post hoc test.

The excessive inflammatory responses trigger activation of astrocytes in an injured spinal cord, which forms a glial scar. This can cause permanent paraplegia by blocking axon regrowth [14]. To determine whether D-GSH provided stronger inhibition of the glial scar formation than L-GSH, we undertook immunohistochemical assessment of TNF-α and glial fibrillary acidic protein (GFAP) in an injured spinal cord (Figure 3A–I). The GFAP fluorescence intensities in the vehicle group and L-GSH group were detected at 14.76 ± 3.47 and 11.44 ± 1.22, respectively (Figure 3J). However, the GFAP fluorescence intensity in the D-GSH group showed a significant decrease compared with that in the vehicle group (D-GSH group: 4.16 ± 1.61, ** *p* < 0.01). TNF-α fluorescence intensity levels in the vehicle group and L-GSH group were detected at 14.72 ± 1.47 and 5.04 ± 2.45, respectively (Figure 3K). However, the TNF-α fluorescence intensity in the D-GSH group was significantly decreased compared with that in the vehicle group (D-GSH group: 0.32 ± 0.19, ** *p* < 0.01). In addition, the TNF-α fluorescence intensity in the D-GSH group was significantly decreased compared with that in the L-GSH group (* *p* < 0.05). Taken together, these results indicate that D-GSH provides stronger suppression of glial scar by decreasing the production of TNF-α and GFAP compared with L-GSH.

### 2.4. D-GSH Reduced Cystic Cavity after SCI

**Figure 4:**
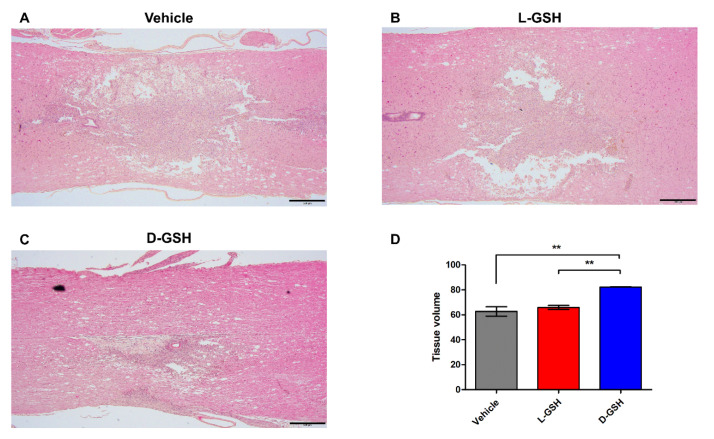
Histopathological changes were evaluated for the (**A**) vehicle-, (**B**) L-GSH-, and (**C**) D-GSH-treated rats. Representative images of the spinal cord stained with H&E in the vehicle, L-GSH, and D-GSH groups. (**D**) Quantitative analysis of the tissue volume. Results are the mean ± standard error of the mean (SEM), ** *p* < 0.01; one-way ANOVA with Tukey post hoc test.

To investigate potential molecular mechanisms underlying this axon regrowth, we examined secondary damage after SCI in rats. The lesion center in the vehicle group indicated the presence of expanded cavity spaces (Figure 4A). The lesion center in the L-GSH group was also observed in the cavity spaces (Figure 4B). However, cystic cavities in the D-GSH group were markedly decreased around the lesion center (Figure 4C). The quantified tissue volume was increased in the D-GSH group compared with that in the vehicle group (Figure 4D, vehicle group: 62.69 ± 6.58, D-GSH group: 82.25 ± 0.40, *** *p* < 0.001). In particular, the tissue volume in the D-GSH group was significantly increased compared with that in the L-GSH group (L-GSH group: 65.92 ± 2.74, ** *p* < 0.01). Our findings show that D-GSH limited the spread of cystic cavities, demonstrating that D-GSH suppresses secondary damage by reducing cystic cavities.

### 2.5. D-GSH-Induced Axon Regrowth across the Lesion Center after SCI

**Figure 5:**
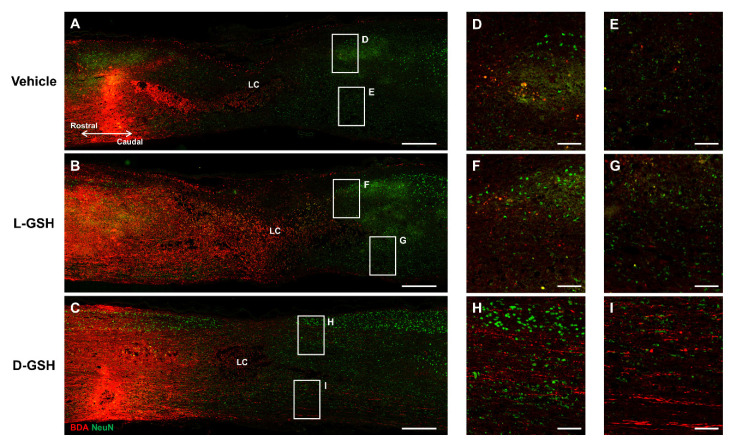
BDA-labelled axons in composite tiled scans of sagittal sections stained for neuron (anti-NeuN, Green), axon (anti-streptavidin, Red). LC, lesion center. Representative images of the (**A**) vehicle group, (**B**) L-GSH group, and (**C**) D-GSH group after SCI in rats. (**D**,**E**) Higher magnification of boxed areas past the lesion center from (**A**). (**F**,**G**) Higher magnification of boxed areas past the lesion center from (**B**). (**H**,**I**) Higher magnification of boxed areas past the lesion center from (**C**). Scale bar, 500 μm (**A**–**C**) and 100 μm (**D**–**I**).

For a more in-depth comparison of the neurological differences caused by the chirality of GSHs, we examined axon regrowth using an anterograde tract-tracer (biotinylated dextran amine, BDA; Figure 5A–C). Axons in the vehicle group were mostly not present caudal to the lesion (Figure 5D,E). Axons in the L-GSH group also were mostly not seen past the lesion center (Figure 5F,G). However, axons in the D-GSH group were extensively observed across the lesion center (Figure 5H,I). Specifically, total axon regrowth across the lesion center in the D-GSH group was over 4-fold greater than that in the vehicle or L-GSH group (Figure S1, vehicle group: 1.95 ± 0.85, L-GSH group: 2.28 ± 1.69, D-GSH group: 8.62 ± 0.43, *** *p* < 0.001).

Afterwards, we evaluated whether increasing axon regrowth could essentially improve functional recovery after SCI in rats (Figure S2). The motor function was assessed on 1, 3, 7, 13, 17, and 21 days post-injury (DPI) using the Basso, Beattie, and Bresnahan (BBB) hindlimb locomotor rating scale [16]. On 1 DPI, there were no differences in the extent of locomotor dysfunction in the vehicle or L-GSH group. However, the locomotor scores of the D-GSH group were significantly increased compared with those of the vehicle and L-GSH group (D-GSH group: 4.00 ± 1.41, ** *p* < 0.01, ## *p* < 0.01). On 3 DPI and 7 DPI, motor functions of the D-GSH group were also significantly improved compared with those of the vehicle and L-GSH group (3 DPI: vehicle group: 0.25 ± 0.50, L-GSH group: 0.75 ± 0.96, D-GSH group: 9.00 ± 2.00; 7 DPI: vehicle group: 1.75 ± 1.50, L-GSH group: 2.00, D-GSH group: 13.50 ± 1.29, *** *p* < 0.001, ### *p* < 0.001). The locomotor scores of the D-GSH group reached a plateau on 7 DPI at 13 points when the locomotor scores of the vehicle group and the L-GSH group reached a plateau on 13 DPI at 3 points and 7 points, respectively.

Our results showed that axon regrowth and motor function was greater in the D-GSH group than in the L-GSH group, indicating that axon regeneration ability and functional recovery differed significantly according to the chirality of GSH.

### 2.6. Chiral GSH Suppressed Inflammation through the Mitogen-Activated Protein Kinase (MAPK) Signaling Pathway after SCI

**Figure 6:**
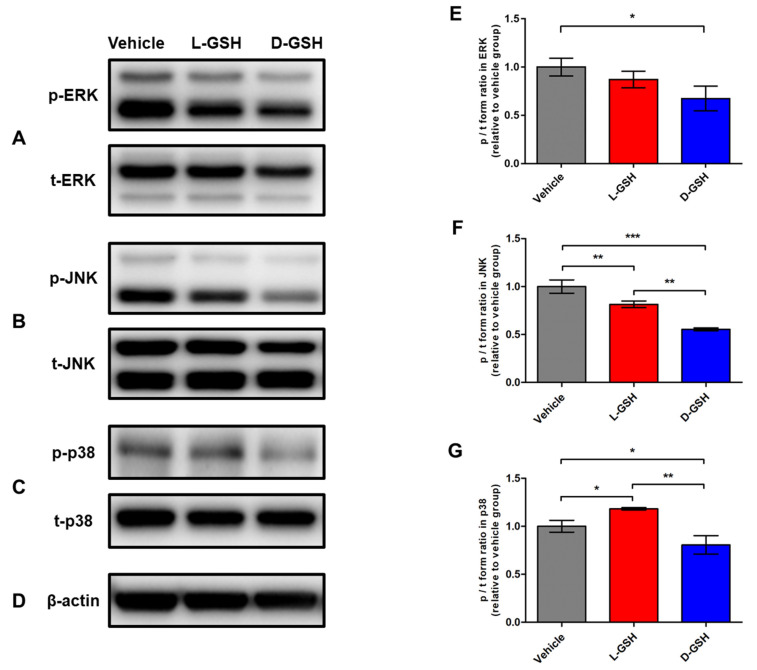
The phosphorylation activities of the MAPK signaling pathway in the vehicle, L-GSH, and D-GSH groups. Representative images of the p and t forms of (**A**) ERK, (**B**) JNK, (**C**) p38, and (**D**) β-actin. Quantitative analyses of the p/t forms of (**E**) ERK, (**F**) JNK, and (**G**) p38. The p/t form volume in the vehicle group was set to 1-fold, and the ratio was relatively calculated and quantified. Results are the mean ± SEM; * *p* < 0.05, ** *p* < 0.01, and *** *p* < 0.001; one-way ANOVA with Tukey post hoc test.

To examine the anti-inflammatory pathway of the chiral GSHs, we examined the activation of the MAPK pathway by Western blotting (Figure 6A–D). The phosphorylated forms of the extracellular signal-regulated kinase (p-ERK), c-Jun N-terminal kinase (p-JNK), and p38 (p-p38) signals in the MAPK pathway are the principal processes that function during the inflammatory response. The fold ratio of the vehicle group was set to 1-fold, and the relative fold change was calculated (Figure 6E–G). The p/t form volume of ERK in the L-GSH group was not lower compared with that in the vehicle group (L-GSH group: 0.87 ± 0.09). However, the p/t volume of ERK in the D-GSH group was decreased as compared with that in the vehicle group (D-GSH group: 0.67 ± 0.13, * *p* < 0.05). The p/t volume of JNK in the L-GSH group was decreased as compared with that in the vehicle group (L-GSH group: 0.81 ± 0.03, ** *p* < 0.01). However, the p/t volume of JNK in the D-GSH group was decreased as compared with those in the vehicle and L-GSH groups (D-GSH group: 0.55 ± 0.01, ** *p* < 0.01, *** *p* < 0.001). Interestingly, the p/t volume of p38 in the L-GSH group was increased as compared with that in the vehicle group (L-GSH group: 1.18 ± 0.01, * *p* < 0.05). On the other hand, the p/t volume of p38 in the D-GSH group was also decreased as compared with those in the vehicle and L-GSH groups (D-GSH group: 0.81 ± 0.10, * *p* < 0.05, ** *p* < 0.01).

As shown in Figure 6, phosphorylation of ERK, JNK, and p38 in the D-GSH group decreased more than those in the L-GSH group. These findings emphasize the different anti-inflammatory effects of chiral GSHs.

### 2.7. Different Uptake Levels of Chiral Gshs in Immune Cells

We hypothesized that the anti-inflammatory effect of GSH may be due to a different uptake level of L- or D-GSH. To test this hypothesis, we investigated different uptake levels of chiral GSHs in activated macrophages by using UV-visible spectroscopy. We calculated the absorbance of various GSH concentrations in cell-free culture medium. A declining tendency was observed at 560 nm according to an increase in the GSH concentration (Figure S3A). From this measurement, we made a calibration standard curve. Then, we treated lipopolysaccharide-stimulated bone marrow-derived macrophages (BMDM) with L- or D-GSH for 24 h and analyzed the GSH uptake ratio by measuring the residual GSH in supernatant. The fold ratio of the L-GSH group was set to 1-fold, and the relative fold change was calculated. Interestingly, the GSH uptake ratio in the D-GSH-treated cells was significantly higher than that in the L-GSH-treated cells (Figure S3B; D-GSH group: 1.51 ± 0.07, *** *p* < 0.001).

## 3. Discussion

In an injured spinal cord, macrophages are differentiated into M1 or M2 macrophages. The number of polarized M1 macrophages peaks at one week after injury and exacerbates neuroinflammation by secreting inflammatory cytokines such as TNF-α and IL-6 [17]. Polarized M2 macrophages induce anti-inflammatory cytokines and growth factors [18]. Astrocytes, the major glial cell type in the CNS, are important contributors to inflammatory immune responses. They produce several neurotrophic substances that regulate the viability of neurons after injury, but they are also a source of pro-inflammatory cytokines such as TNF-α, IL-1β, and IL-6 [19]. Activated astrocytes produce a GFAP and form a glial scar, which blocks axon regrowth in the CNS. We observed that D-GSH significantly decreased inflammatory cytokines and glial scar formation compared with L-GSH. This indicates that D-GSH suppressed astrocyte glial scar by suppressing differentiation into M1 macrophages more compared with that by L-GSH.

Severed axons in the injured spinal cord do not transmit nerve impulses. Macaya and Spector reported that the increased tissue repairing around cavity spaces induced axon regrowth [20]. Moreover, Hong et al. found that increased axon regeneration was accompanied by the preservation of motor neurons and functional recovery [21]. In line with these findings, D-GSH-treated rats exhibited axon regrowth across the lesion center and a decrease in cystic cavity formation. Of note, quantified total axon regrowth across the lesion center in the D-GSH-treated rats was over 4-fold greater than that in the vehicle or L-GSH-treated rats.

Inflammatory responses are typically triggered by major intracellular signaling pathways of the MAPK pathway, including ERK, JNK, and p38 [22]. JNK plays an essential role in regulating immune responses. Especially, ERK and p38 are determinants of neuronal survival on certain occasions [23]. Specifically, activation of the MAPK pathway increases the production of interferon and inflammatory cytokines [24], leading to the migration of macrophages [25]. We identified that D-GSH inhibited phosphorylation of ERK, JNK, and p38 signaling more than L-GSH. Collectively, our findings demonstrate the different mechanisms of the CNS immune system through the chirality of GSHs. After SCI, nitric oxide (NO) radicals and superoxide radicals form peroxynitrite [26], inducing vascular relaxation [27]. This accelerates the pro-inflammatory response by increasing the infiltration of activated macrophages [28]. GSH plays a major role in maintaining redox homeostasis and in the direct scavenging of NO radicals owing to its high concentration in cells [29]. Recently, Yeom et al. demonstrated that D-chirality had higher cellular uptake than l-chirality in the bio-systems [11]. In light of taking together those earlier results, our study suggests that D-GSH can decrease the inflammatory response, given its higher uptake by activated macrophages compared with L-GSH depending on its chirality.

## 4. Materials and Methods

### 4.1. Preparation and Characterization of Chiral Gshs

L-GSH was purchased from Sigma-Aldrich (St. Louis, MO, USA), and D-GSH was obtained from KOMA Biotech (Seoul, Korea). The chirality of the GSHs was measured by CD spectroscopy (Chirascan-plus, Applied Photophysics, Leatherhead, UK). For the in vivo experiment, L- or D-GSH was dissolved to a concentration of 10 mM using 0.9% sterile saline in each case.

### 4.2. Surgical Procedures

Experiments were conducted on seven-month-old female Sprague Dawley rats (170–210 g body weight). Twenty-seven rats were divided into a vehicle group (*n* = 9), an L-GSH group (*n* = 9), and a D-GSH group (*n* = 9).

For SCI modeling, the rats were anesthetized through a Zoletil^®^ (50 mg/kg, Virbac Laboratories, Carros, France)/Rompun^®^ (10 mg/kg, Bayer, Seoul, Korea) solution administered intraperitoneally. Then, a midline incision was made in the lower back. To expose the T9, tissues were dissected layer by layer to reveal the T8–T10 vertebra. A T9 total laminectomy was conducted to expose the dura. Vertebral column was supported and stabilized by clamps at the T7 and T11 spinous processes. A stainless-steel rod weighing 40 g (2.5 mm in diameter) was dropped from a height of 30.5 mm onto the dorsal surface of the spinal cord to induce injury using an impactor (RWD, CA, USA, Model: 68097) [30]. After the injury, the weight rod was quickly removed. Each treatment was applied stereotaxically onto the SCI lesions 1 mm below the surface at 1 μL per minute for five minutes. Each dose was repeated three times around the lesion center. After the injury, the surgical site was closed layer by layer. Tract-tracing of axons was performed via BDA injections (Sigma, St. Louis, MO, USA; #D1956). One week after injury, the BDA injections were performed to three SCI rats from each group. BDA was injected into two sites (one on each side of the cord, 0.5 μL (dissolved in sterile saline) 1.5 mm below the surface at 0.1 μL per minute using a 33-gauge Hamilton syringe. After surgery, the rats were kept warm and housed separately, with free access to food. On each day at 8 a.m. and 8 p.m., a bladder massage was conducted to assist with urination. All surgeries were performed by the same spine neurosurgeon (S. Sohn).

### 4.3. Hindlimb Locomotor Evaluation

To test the hindlimb function, an open-field locomotion was evaluated using BBB scores [16]. After SCI, BDA-injected rats were evaluated on 1, 3, 7, 13, 17, and 21 DPI. Two trained investigators who were blind to the experimental conditions performed the behavioral analyses.

### 4.4. Histology and Immunohistochemistry

Seven days after SCI, three rats from each group were anesthetized and perfused transcardially with 4% paraformaldehyde. Three BDA-injected rats from each group were sacrificed 21 days after SCI. The spinal cords were exposed from the original incisions in the back. Centered at the injury site, a 10 mm segment of the spinal cord was dissected, embedded in paraffin, and sectioned for histological analysis. For the quantification of the H&E staining, serial sagittal sections through the dorsoventral axis of the spinal cord (5 μm thick) were collected at lengths of every 100 μm (20th), 150 μm (30th), and 200 μm (40th) per rat. The sections for each rat were stained with H&E solution (BBC Biochemical, Mount Vernon, WA, USA) and analyzed. At each section, the most severely injured site of the spinal cord around the center was selected as the region of interest (ROI, 1500 μm^2^ × 1000 μm^2^). The morphological changes of the H&E stained tissues were observed at ×40 (scale bar: 500 μm) and ×100 (scale bar: 200 μm) magnification using a light microscope (Olympus IX71, Tokyo, Japan). A designated rectangular area as the ROI represented 100%. The cavity tissue volume was calculated and quantified using ImageJ software (NIH). Immunofluorescence was processed as described in the literature [31]. For the quantification of the BDA fluorescence intensities, serial sagittal sections through the dorsoventral axis of the spinal cord (5 μm thick) were collected every 105 μm (21st), 155 μm (31st), and 205 μm (41st) from three rats per group/pre-determined day. We designated three rectangular areas (489.16 μm × 581.19 μm) across the lesion center in composite tiled scan images (6900 μm × 2500 μm) for each group. The area of the captured images under ×400 magnification was 420,000 (600 × 700) pixels. The stained immunofluorescence pixels per image were calculated and quantified using the intensity measurement method available in the ImageJ software package (NIH). Primary antibodies were as follows: rabbit anti- GFAP (1:1000), mouse anti-TNF-α (1:1000), and rabbit anti-NeuN (1:1000), all from Abcam. Fluorescence secondary antibodies were conjugated to Alexa 488 or Alexa 568 (Molecular Probes). BDA tract-tracing was visualized with streptavidin-Alexa 594 (1:1000, Thermofisher). The sections were mounted using a fluorescence mounting medium (DAKO, Glostrup, Denmark). Sections were examined and photographed using light microscopy (DP74, Olympus, Tokyo, Japan) or confocal laser scanning microscopy (LSM 880, Carl Zeiss, Jena, Germany).

### 4.5. Quantification of Inflammatory Cytokines

Seven days after SCI, three rats from each group were sacrificed to collect segments of the spinal cord (10 mm) including the lesion center. The segments were then washed with phosphate-buffered saline and homogenized in lysis buffer. Afterwards, they were centrifuged at 13,000 rpm at 4 °C for 15 min. Protein concentrations in the tissue lysates were measured with the aid of a bicinchoninic acid protein assay kit (Thermo Scientific, Rockford, IL, USA). TNF-α, IL-1β, IL-6 (Koma Biotech, Seoul, Korea), and COX-2 (CUSA bio, Wuhan, China) enzyme-linked immunosorbent assay (ELISA) kits were used according to each manufacturer’s directions.

### 4.6. Western Blotting

Equal amounts of protein (30 μg) were subjected to sodium dodecyl sulfate-polyacrylamide gel electrophoresis and transferred to nitrocellulose membranes. The membranes were incubated with 5% bovine serum albumin for one hour to block the nonspecific binding and probed with primary antibodies with the p-ERK (1:1000), p-JNK (1:1000), and p-p38 (1:1000). Subsequently, equal membranes were stripped and reprobed with the total forms of ERK (t-ERK; 1:1000), JNK (t-JNK; 1:1000), and p38 (t-p38; 1:1000). All primary antibodies were purchased from Cell Signaling Technology (Danvers, MA, USA), except for β-actin (1:5000; Abcam, Cambridge, UK). As an internal control, β-actin was probed into the membranes. The primary antibodies were followed by incubation with secondary antibodies (1:5000; Santa Cruz Biotechnology, Dallas, TX, USA). The visualized signal bands were detected using an ECL solution (Amersham, Buckinghamshire, UK) through a ChemiDoc XRS System (Bio-Rad, Hercules, CA, USA). The phosphorylated form/total form (p/t form) volumes were calculated and quantified using ImageJ software. The p/t form volume of the vehicle group was set to 1-fold, and the ratio change of the normalized fold was relatively calculated and quantified.

### 4.7. Isolation of Bone Marrow-Derived Macrophages

The isolation protocol from rat bone marrow by Virginie et al. was used [32]. Briefly, rats were euthanized by CO_2_ asphyxiation and the femur and tibia were collected aseptically. Each bone was cut at the joint. Bone marrow was collected by rinsing the bone inner cavity with Dulbecco’s modified Eagle’s medium (DMEM) containing 10% fetal bovine serum (FBS), 2% glutamate, and 1% penicillin. The collected bone marrow was centrifugated (10 min, 450× *g*). Erythrocytes were lysed via red blood lysis buffer (Sigma, St. Louis, MO, USA; #37757) for 30 s. Then, DMEM was quickly added to the cells, after which they were centrifuged. To eliminate resident macrophages, the cells were filtered with a 40 μm membrane and were incubated for four hours at 37 °C in tissue-culture-treated plates. Afterwards, supernatants were collected and centrifuged, and the pellet was dissociated in 150 mL of a medium consisting of complete DMEM (cDMEM) containing 10% FBS, 2% glutamate, 1% penicillin, and 20 ng/mL recombinant rat macrophage colony-stimulating factor (#315-02; Peprotech, Rocky Hill, NJ, USA). Cells were distributed at 10 mL of suspension per Petri dish (15 Petri dishes; Corning, #430591) and cultivated at 37 °C and 5% CO_2_. After three days, we added 10 mL of cDMEM to each Petri dish. The dishes were incubated for four additional days. At seven days, the cells of BMDM were harvested and seeded for the indicated experiments.

### 4.8. Calculation of Intracellular Uptake Rates

The absorbance levels of various GSH concentrations (0, 0.15625, 0.3125, 1.25, 2.5, 5, and 10 mM) were calculated by UV-visible spectroscopy. These values formulated the GSH standard curve. Then, BMDM cells were seeded on a Petri dish at a density of 1 × 107 cells/dish and treated with 10 mM L- or D-GSH for 24 h. The absorbance of each supernatant was measured by a microplate reader (Bio-Rad, Hercules, CA, USA) at a wavelength of 560 nm. Afterwards, we analyzed the uptake ratios based on the GSH standard curve. The fold ratio of the L-GSH group was set to 1-fold, and the relative fold change was calculated.

### 4.9. Statistical Analyses

Analysis of variance (ANOVA) was used to compare differences in the BBB score at different time points. Two-group comparisons were conducted with Student’s *t*-Tests. Differences with *p*-values of * *p* < 0.05, ** *p* < 0.01, and *** *p* < 0.001 were considered to be statistically significant.

## 5. Conclusions

In this study, D-GSH increased axon regrowth past the lesion site and improved functional recovery by suppressing secondary damage. Furthermore, D-GSH inhibited inflammatory cytokines, suppressed the MAPK signaling pathway, and showed more interaction with activated macrophages than L-GSH. Although the anti-inflammatory potential of L-GSH has been reported for other anatomical sites [33,34,35], to the best of our knowledge, this is the first report to investigate the chirality-dependent anti-inflammatory effects of chiral GSHs after SCI in an animal model. Our findings suggest that chiral GSH can serve as an alternative drug for SCI. It may also have therapeutic potential as a treatment for other diseases.

## Data Availability

Data are contained within the article and Supplementary Materials.

## Supplementary Materials

The following are available online at https://www.mdpi.com/article/10.3390/ph14080792/s1, Figure S1: Quantitative analyses of BDA-labelled axons past the lesion center in the vehicle, L-GSH, and D-GSH groups are shown, Figure S2: Comparison of BBB locomotor scores in the vehicle, L-GSH, and D-GSH groups, Figure S3: Comparative evaluation of the intracellular uptake ratios of the chiral GSHs.
