# Supplementary material for: Chirality-Dependent Anti-Inflammatory Effect of Glutathione after Spinal Cord Injury in an Animal Model
Source: Pharmaceuticals (Basel). 2021 Aug 12;14(8):792. doi: 10.3390/ph14080792 (PMC8398565; doi:10.3390/ph14080792)
Supplement: Supplementary file 1 [file pharmaceuticals-14-00792-s001.zip › pharmaceuticals-1297759-supplementary.pdf]

Supplementary Materials for

Chirality-dependent anti-inflammatory effect of glutathione after spinal cord injury in an animal model

Seong-Jun Kim [1], Wan-Kyu Ko [1], Gong-Ho Han [1], Daye Lee [1], Yuhan Lee [2], Seung-Hun Sheen [3], Je-Beom Hong [4], Seil Sohn [3,*]

[1] Department of Biomedical Science, CHA University, Seongnam-si, Gyeonggi-do, 13493, Republic of Korea; ksj987456@chauniv.ac.kr (S.J.K.); wankyu@chauniv.ac.kr (W.-K.K.); hgh429@chauniv.ac.kr (H.G.H.); day03@chauniv.ac.kr (D.L.).

[2] Department of Medicine, Brigham and Women's Hospital, Harvard Medical School, Boston, MA, 02115, USA; ylee21@bwh.harvard.edu (Y.L.).

[3] Department of Neurosurgery, CHA Bundang Medical Center, Seongnam-si, Gyeonggi-do, 13496, Republic of Korea; nssheen@cha.ac.kr (S.H.S.); sisohn@cha.ac.kr (S.S.).

[4] Department of Neurosurgery, Kangbuk Samsung Hospital, Sungkyunkwan University School of Medicine, Seoul, 03181, Republic of Korea; jebeomhong@gmail.com (J.B.H.).

[*] Corresponding author: sisohn@cha.ac.kr; Tel: +82-31-881-7966

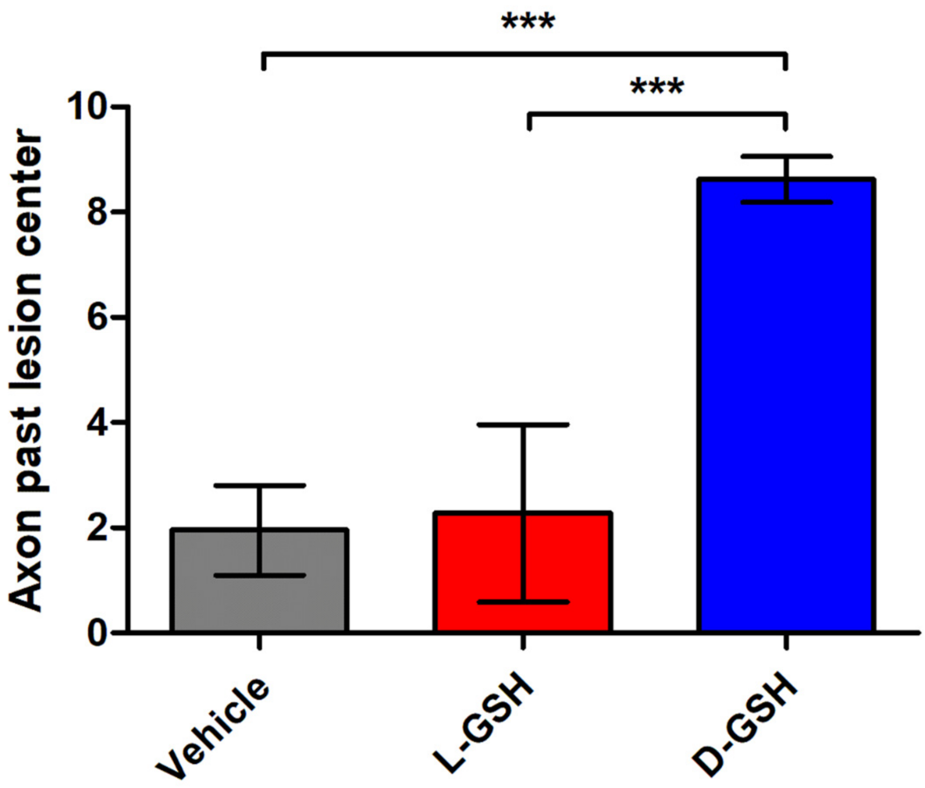

Figure S1. Quantitative analyses of BDA-labelled axons past the lesion center in the vehicle, L-GSH, and D-GSH groups are shown. Results are the mean ± SEM; $^*p < 0.05$, $^{**}p < 0.01$, and $^{***}p < 0.001$; one-way ANOVA with Tukey post-hoc test.

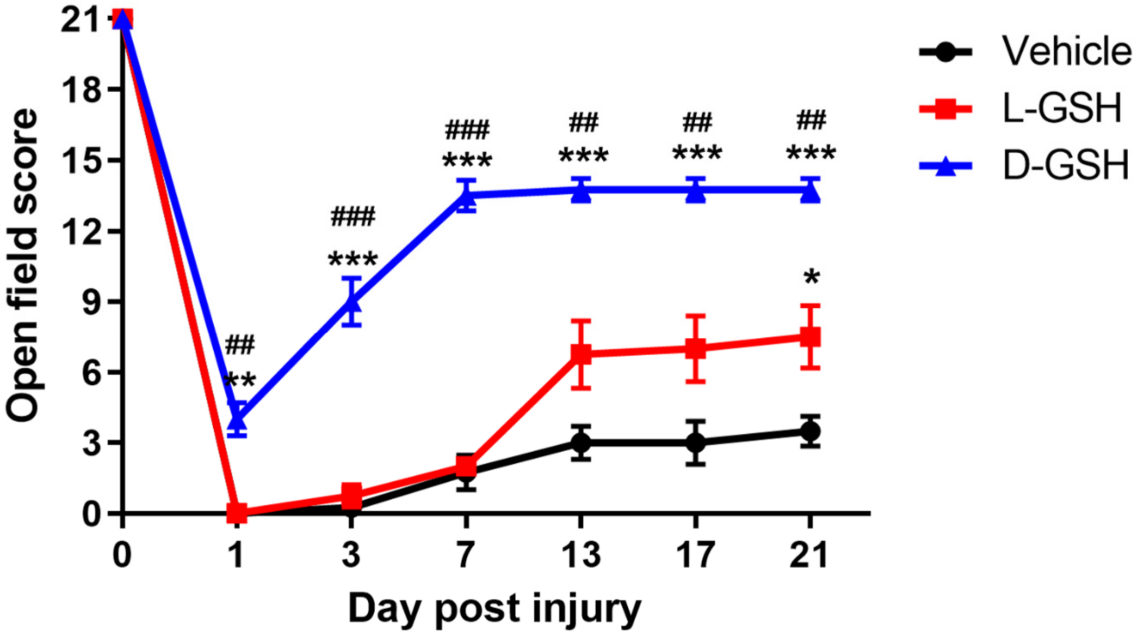

Figure S2. Comparison of BBB locomotor scores in the vehicle, L-GSH, and D-GSH groups. The BBB scores were evaluated for 21 days after SCI. * denotes a significant difference between the vehicle group and the D-GSH group. # denotes a significant difference between the L-GSH group and the D-GSH group. Results are the mean ± standard deviation (SD); *$p < 0.05$, **p and ##$p < 0.01$, and ***p and ###$p < 0.001$. Unpaired two-tailed Student's t-tests.

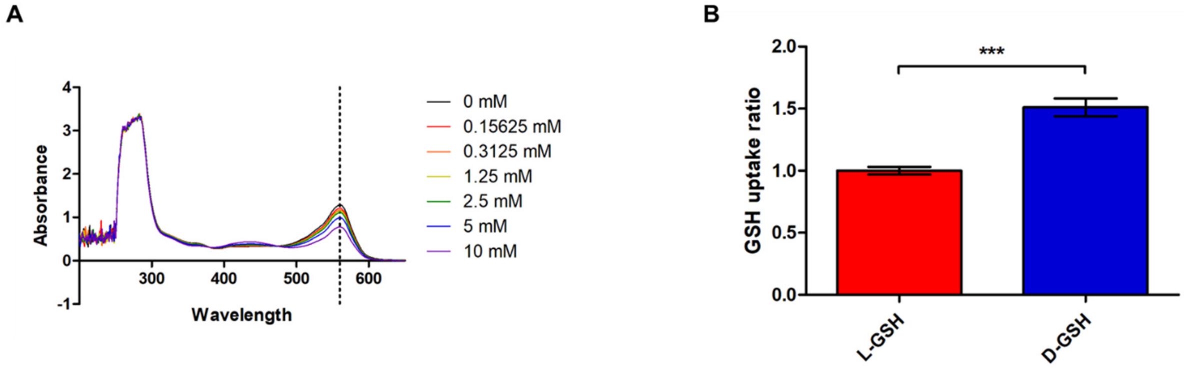

Figure S3. Comparative evaluation of the intracellular uptake ratios of the chiral GSHs. **(A)** Absorbance of various GSH concentrations. **(B)** Intracellular uptake ratios of macrophages treated with L- or D-GSH based on (A). The fold ratio of the L-GSH group was set to 1-fold and the relative fold change was calculated. Results are the mean ± SEM; $^{*}p < 0.05$, $^{**}p < 0.01$, and $^{***}p < 0.001$; one-way ANOVA with Tukey post-hoc test.
